# Histologic evaluation of furcation perforation treated using bioceramic putty with and without platelet rich fibrin or chitosan hydrogel as an internal matrix

**DOI:** 10.1038/s41598-025-20663-w

**Published:** 2025-09-30

**Authors:** Muhammad Salah-Uddin Anwar Laithy, Abeer Mostafa Darrag, Neveen Ali Shaheen, Khaled Mohammed Ali, Sarah Yasser AbuAli

**Affiliations:** 1 Department of Endodontics, Faculty of dentistry, Tanta University, Tanta, Egypt; 2 Department of Surgery, Anesthesiology and Radiology, Faculty of Veterinary Medicine, Cairo University, Cairo, Egypt; 3 Department of Oral Biology, Faculty of dentistry, Tanta University, Tanta, Egypt; 4 Department of Oral Biology, College of Oral and Dental Medicine, Alsalam University, Tanta, Egypt

**Keywords:** Chitosan hydrogel, Furcation perforation repair, Internal matrix, Platelet-rich fibrin, Premixed BC putty, Health care, Pathogenesis

## Abstract

The present study investigated the tissue reaction of platelet rich fibrin and chitosan hydrogel as internal matrices in repairing furcal perforations in mature dogs’ teeth. Seventy-two teeth in six mongrel dogs were experimented in this study. After access opening, root canal preparation was completed and obturation was done using gutta percha/resin sealer. Furcation perforations were done, and the experimental teeth were classified according to the perforation repair protocol to three experimental groups and a positive control group (18 teeth each). Group 1: Platelet-rich fibrin matrix with premixed calcium silicate-based bioceramic putty (BC putty), Group 2: Chitosan hydrogel matrix with BC putty, Group 3: BC putty alone and Group 4: a positive control group where no repair material was utilized. Access openings were restored with composite filling. The experimented teeth and the supporting bone were sectioned into blocks and histologically examined for tissue reaction at one and three months. Statistical analysis was performed using Chi-square test, where the significance level was set at *P* ≤ 0.05. BC putty and BC putty with PRF matrix exhibited less bone loss, epithelial proliferation and inflammatory reaction compared to chitosan hydrogel at one and three months intervals, also they showed more hard tissue deposition compared to chitosan hydrogel at 3-month interval. Although BC putty presented higher sealing ability with great area of newly formed hard tissue compared to chitosan hydrogel, BC putty with PRF can be considered as a successful management option for furcal perforation repair. Management of perforation is considered a challenging procedure especially when located in the furcation area, however histological evaluation of the tissue reaction to different internal matrices materials could provide favorable clinical outcomes concerning the perforation repair procedures.

## Introduction

Furcation perforation is described as a pathologic continuation between the pulp space and the outer tooth surface in the pulpal floor of furcated teeth. The aim of repairing furcation perforation is to close the defect present in the dentin and establish good condition for new periodontal reattachment. Since the furcation perforation involves dental and periodontal tissues, each tissue type within the perforation defect should be considered separately.

The use of artificial floor technique was suggested for repairing furcal perforations dealing with the periodontal and dentinal wounds as separate entities^1^. Several authors stated that calcium sulfate when placed below glass ionomer to repair furcation perforations it was stable, biocompatible and shows resorption rate in accordance with that of new hard tissue formation. In addition, it prevents epithelial cells from invading the site of new hard tissue formation allowing the periodontal reattachment^2^.

Platelet-rich fibrin (PRF) is considered a second generation of platelet concentrates. It is rich in fibrin, growth factors and platelets. It enhances deposition of new bone and increases the healing rate of grafted bone^3^.

Chitosan (CH) polymers are natural aminopolysaccharides obtained by deacetylation of chitin that is present in shells of shrimps. It has a lot of biological properties such as antimicrobial action, high biocompatible properties, and it promotes cell adhesion, proliferation and differentiation^4^. Therefore, it has commonly been used as matrix for tissue engineering^5^.

Premixed bioceramic (BC) material formulations have become available. Well-Root™ PT is a ready-made bioceramic in a putty formulation used for pulp capping, perforation repair, and different surgical applications. It is highly alkaline and is composed of a calcium aluminosilicate, that requires water for setting and hardening. It shows no shrinkage during setting and exhibits excellent physical and biological characteristics. It is also bioactive, so that does not induce tissue inflammatory and enhances mineralization^6^.

Taking into consideration the favorable outcomes of perforation repair procedures, up till now there has been limited currently available literature dealing with the perforation as a heterogenous wound with periodontal and dentinal tissues as separate entities. Therefore, the present experiment aimed to evaluate the tissue response after using different matrix materials in treatment of furcal perforation.

## Methods

### Ethical approval

The Institutional Research Ethics Committee (REC), Faculty of Dentistry, Tanta University, Egypt reviewed and approved this work (R-END-02-22-11). All methods were performed in accordance with the relevant guidelines and regulations. All methods reported are following the ARRIVE guidelines for the animal experiments (https://www.arriveguidelines.org)^7^.

### Sample size calculation

The study comprised 72 specimens, divided equally into four groups (*n* = 18 each), with 9 specimens evaluated at 1 month and 9 at 3 months. The sample size was primarily determined based on feasibility and in line with previous in-vitro studies of similar design^8–10^. To increase transparency, a post-hoc power analysis was performed for the chi-square test using G*Power (version 3.1.9.2; University of Kiel, Germany). With α = 0.05 and df = 3, the achieved power with the present total sample size (*N* = 72) was approximately 0.10 for a small effect (w = 0.10), 0.55 for a medium effect (w = 0.30), and 0.96 for a large effect (w = 0.50). Thus, the study design was adequately powered to detect large effects, but underpowered for small-to-moderate effects. These limitations have been acknowledged. Teeth were randomly separated into four groups (one control and 3 experimental groups) using a web program (http://www.random.org/).

### Animal model selection

Dogs were selected for the present experiment from the animal housing present in Faculty of Veterinary Medicine, Cairo University, Egypt. These dogs were used in this scientific teaching and research purposes because they had already been requested and slated for euthanasia for veterinary reasons. The euthanasia was scheduled for non-health-related institutional reasons (e.g., age, research rotation, housing capacity), rather than due to disease. All efforts were done to decrease the dogs used in the study.

### Preparation of animals

Six mongrel dogs weighing about 12 to 16 kg and more than 1 year old, were chosen for this study. They have 72 upper and lower bifurcated premolars with mature roots. The second, third and fourth upper and lower, right and left premolar teeth in each dog were used (12 teeth/dog, 3 in each quadrant). The dogs were injected with Ivermectin (MSD, Haarlem, Netherland.) 200 mg/KG body weight subcutaneously to guard against external and internal parasites and quarantined in separate cages in the Department of Surgery, Anesthesiology and Radiology, Faculty of Veterinary Medicine, Cairo University, Egypt. Dogs were examined where dogs used for the experiment were fed and followed for two weeks before this study. Diseased dogs were excluded.

### General anesthesia

All dogs were premedicated with 0.05 mg/kg Atropine sulphate (C.I.D., Cairo, Egypt) (1 mg/mL injected subcutaneously and 1 mg/kg Xylazine HCl (ADWIA Co., Cairo, Egypt) injected intramuscularly. General anesthesia was then inducted using Ketamine HCl (EIMC pharmaceuticals Co., Cairo, Egypt) injected through the cephalic vein at a dose of 5 mg/kg body weight. Anesthesia was maintained with 2.5% Thiopental sodium (Sandoz, Kundl, Austria) at a dose of 25 mg/kg body weight injected intravenously^8,9^.

## Operative procedures

### Access cavity Preparation and root canals instrumentation

Prior to the operative procedures, the oral cavity was cleaned and disinfected using povidone-iodine (Betadine, 10%, Mundi Pharma, Cairo, Egypt). Rubber dam was used to isolate the premolar teeth. The enamel and rubber dam material were disinfected with 30% hydrogen peroxide (H_2_O_2_, LUNA pharmaceutical, Cairo, Egypt), and 2.5% sodium hypochlorite (Clorox Co., 10th of Ramadan City, Egypt).

After rubber dam application, access openings were prepared through the occlusal aspect of the selected premolars using a small round bur (Mani Inc, Touchigi-Ken, Japan.).

The working length was then measured to be within 2 mm from the radiographic apex^10^. Preparation of the root canals was completed with manual nickel-titanium K-files using a step-back technique. Root canals were then dried and obturated with gutta percha (DiaDent, Chongju City, Korea.) and resin sealer (Meta Biomed, Chungcheongbuk-do, South Korea.) using cold lateral compaction technique.

### Furcation perforation creation

A perforation of 1.4 mm diameter was done centralized in the pulpal floor using a 1.4 mm diameter (ISO size 014) low speed, long shank, rose head bur (Hager & Meisinger GmbH Hansemannstr, Neuss, Germany). The perforation depth was adjusted to be 2 mm limited into the interradicular bone^11^. The perforation sites were irrigated using a 3 mL of normal saline. Sterile gauze was used to swab and control the bleeding.

### Study groups for perforation repair

The teeth were classified depending on the perforation repair protocol to three experimental groups and a positive control group (18 teeth in each group) (*n* = 72 teeth/ 6 dogs). In each dog, all groups were represented where every group was performed in a quadrant as follows:

#### Group 1

Platelet-rich fibrin matrix and base of Well-Root™ PT (Vericom, Gangwon-Do, Korea.).

PRF matrix was obtained by taking a 10 mL blood sample from the Jugular vein without adding anticoagulant in 10 mL test tube and centrifuged immediately at about 2700–3000 rpm for ten-twelve minutes in centrifuge machine ^(^Laboratory PRF, PRP tube box dental centrifuge, Manson Inc, Beijing, China.)^3^. The PRF clot was held from the tube with a sterilized tweezer and then pressed between sterile gauze pieces. The squeezed PRF was cut into pieces using a scalpel blade.

**Fig. 1:**
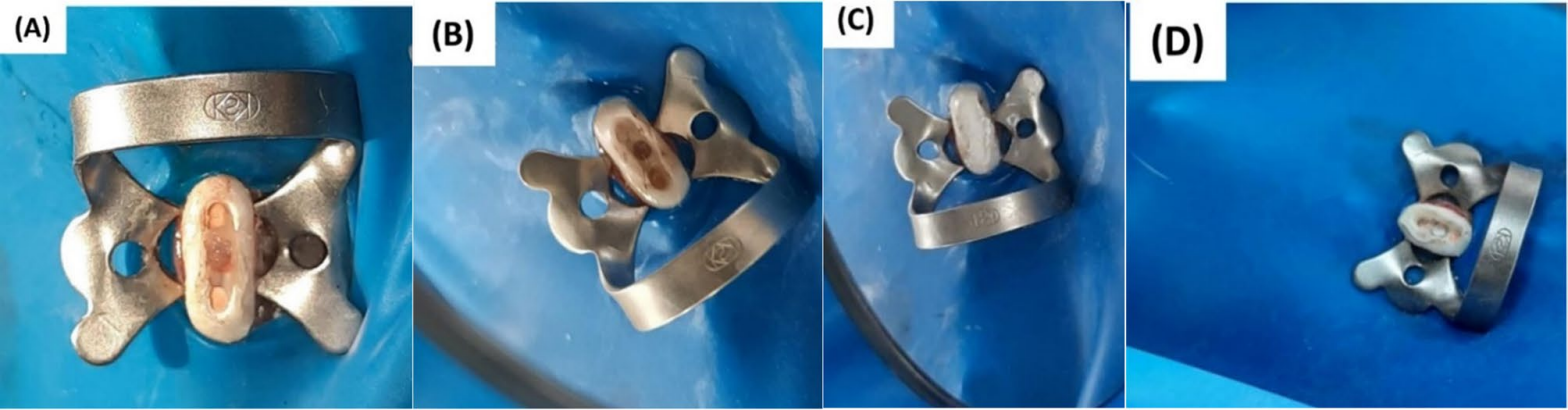
Representative clinical picture for each group (**A**) PRF piece sealing the furcation perforation site. (**B**) Chitosan hydrogel in the perforation site. (**C**) Well-Root™ PT was placed directly to seal the perforation with no matrix used. (**D**) Positive control group perforation defect sealed by Teflon.

**Fig. 2:**
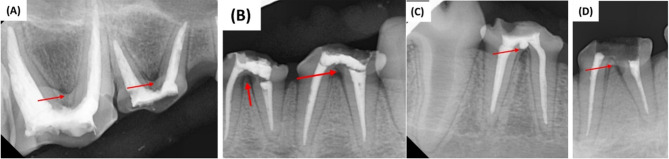
Representative postoperative radiograph for each group after placing the Well-Root™ PT(**A**) PRF matrix in the perforation (arrow). (**B**) Chitosan hydrogel matrix in the perforation (arrow). (**C**) Well-Root™ PT placed with no matrix used in the perforation (arrow) (**D**) Positive control group where no repair material used in the perforation (arrow).

The PRF pieces were carried into the root furcation perforation sites, gently placed into the perforations and adapted with a hand plugger (Fanta Dental Materials Co., Shanghai China.) (Fig. 1). After placing of the PRF, Well-Root™ PT capsule was loaded into a special gun (Huanghua Promisee Dental Co., Huanghua, China.) and delivered to the pulpal floor and then compacted carefully using hand plugger as a base of 2–3 mm thickness over the pulp chamber floor and a periapical x-ray was taken to confirm its proper placement before the final restoration (Fig. 2). Finally, the coronal access cavities were closed with composite restoration (Filtek Z250 3 M ESPE, St.Paul, USA.).

#### Group 2

Chitosan hydrogel (Nano Gate, Mokattam city, Egypt.) matrix and base of Well-Root™ PT.

Chitosan hydrogel was freshly prepared based upon previously published protocols by dissolving 200 mg of low molecular weight chitosan polymer in 9 ml acetic acid with concentration of 1% to form a viscous stock solution with a 2% concentration of and this solution was sterilized in autoclave (Tau sterile, Province *of* Como, Italy.) at 121 °C for 20 min^12,13^. The pH of chitosan was 4.8 and was elevated to 7 by adding 32 mmol/L b-glycerophosphate disodium salt carefully until the solution became clear. Chitosan hydrogel was carried into the root furcation perforation sites using a 3 mL plastic disposable syringe.

After placing the chitosan hydrogel in the perforation site (Fig. 1), Well-Root™ PT was applied, and access cavities were filled as in group1.

#### Group 3

Well-Root™ PT in the perforation.

Well-Root™ PT was placed to fill the perforation directly and then compacted (Fig. 1). The coronal access openings were restored as the previous groups.

#### Group 4

Positive control group with induced perforation where no repair material was used, and the perforation was sealed with sterile Teflon (Fig. 1). The Teflon was sterilized in the autoclave after wrapping it around a tongue depressor in a spiral form. The access openings were restored as the previous groups.

Then the dogs returned to their animal housing and kept under continuous monitoring of weight and food intake. The dogs were given intramuscular Amoxoil retard (LABORATORIES SYVA S.a., Pablo Diez, Spain.) at a dose of 10 mg/kg and Acetaminophen (Pharmaco B International, Borg Al-Arab, Egypt.) at a dose of 1.1 mg/kg once/day for 5 days for controlling pain and infection respectively after completion of the procedure.

### Euthanasia/sacrifice method

After the end of each observation period (1 or 3 months), dogs were euthanized by anesthetic overdose using 40 mL of 2.5% thiopental sodium (Sandoz, Kundl, Austria) injected rapidly into the cephalic vein. Both upper and lower arches were removed surgically.

### Histopathological evaluation

Block sections including each tooth and the supporting bone were obtained. Blocks obtained undergo fixation using a formalin solution of 10% concentration (El Gomhoria Co. for trading pharmaceuticals, SAE, Egypt.). After two weeks of tissue fixation, blocks undergo decalcification using 17% EDTA (Alpha Chemika, Mumbai, India.). EDTA solution was renewed daily for about 120 days. After decalcification, specimens undergo dehydration in 70% ethanol then embedded in paraffin was. Serial sections of 4 μm thickness in a longitudinal manner parallel to the mesiodistal direction passing through the perforation area were made and mounted on glass slides, deparaffinized, hydrated, and stained with hematoxylin and eosin^14^. The stained sections were blindly examined by two experienced observers independently the following parameters:

### Inflammatory cell count

The average inflammatory cell was counted using image analysis software (Image J 1.41a, NIH, USA). Image J software is a powerful image analysis software widely used in biomedical research. To determine the degree of inflammation, five fields of high-power magnification were obtained from the prepared slides. Then cells was counted within the five high-power fields and the average was obtained as described previously by Morse et al.^15^. The inflammatory reaction was grade similar to other studies^16–18^ as follows:

Score zero: presented none or few inflammatory cells (no reaction), score 1: less than 25 cells (mild reaction), score 2: 25–125 cells (moderate reaction) and score 3: more than 125 cells (severe reaction).

### Hard tissue deposition into the perforation

Score 0: absent, score 1: partial and score 2: complete (seal of the perforation).

#### Bone resorption

Score 0: absent and score 1: present.

#### Epithelial proliferation

Score 0: absent and score 1: present.

#### Regeneration of periodontal ligament at the furcation area

Score 0: absent and score 1: present.

### Statistical analysis

Data was collected, tabulated and analyzed using Statistical Packages for the Social Sciences (SPSS) version 21 (IBM, Armonk, NY, USA). Chi-square tests were used to assess differences in categorical outcomes among the four groups at each time point. Where appropriate, pairwise chi-square comparisons with Bonferroni adjustment were conducted to identify inter-group differences. The level of significance was set at *P* ≤ 0.05.

## Results

In this study, the dogs were examined at weekly intervals from the time of operation to the time of sacrifice. Dogs showed no changes in their habits. Also, they showed no signs of pain or suffering and no dogs were excluded from studying.

**Fig. 3:**
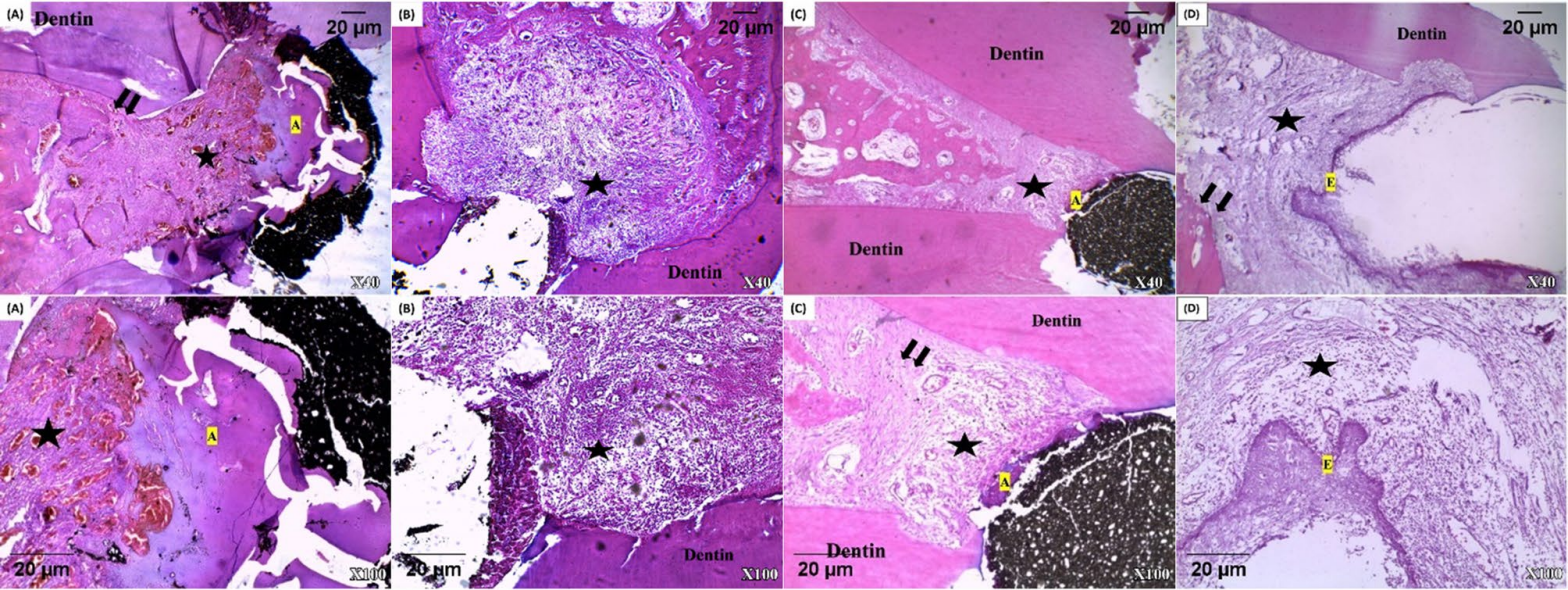
Photomicrographs of all groups after one month. **(A)** PRF group with almost complete bridge of newly formed hard tissue (A) surrounding connective tissue with minimal inflammation (black star) and regeneration of PDLs (black arrows) **(B)** Chitosan hydrogel group where the surrounding connective tissue shows prominent inflammation (black star) **(C)** Bioceramic group with newly formed hard tissue (A) with surrounding connective tissue with signs of inflammation (black star) and regeneration of PDLs (black arrows) **(D)** Positive control group with epithelial invasion (E) overlying inflamed connective tissue (black star) with bone resorption (black arrows) (H-E; X40-100).

**Fig. 4:**
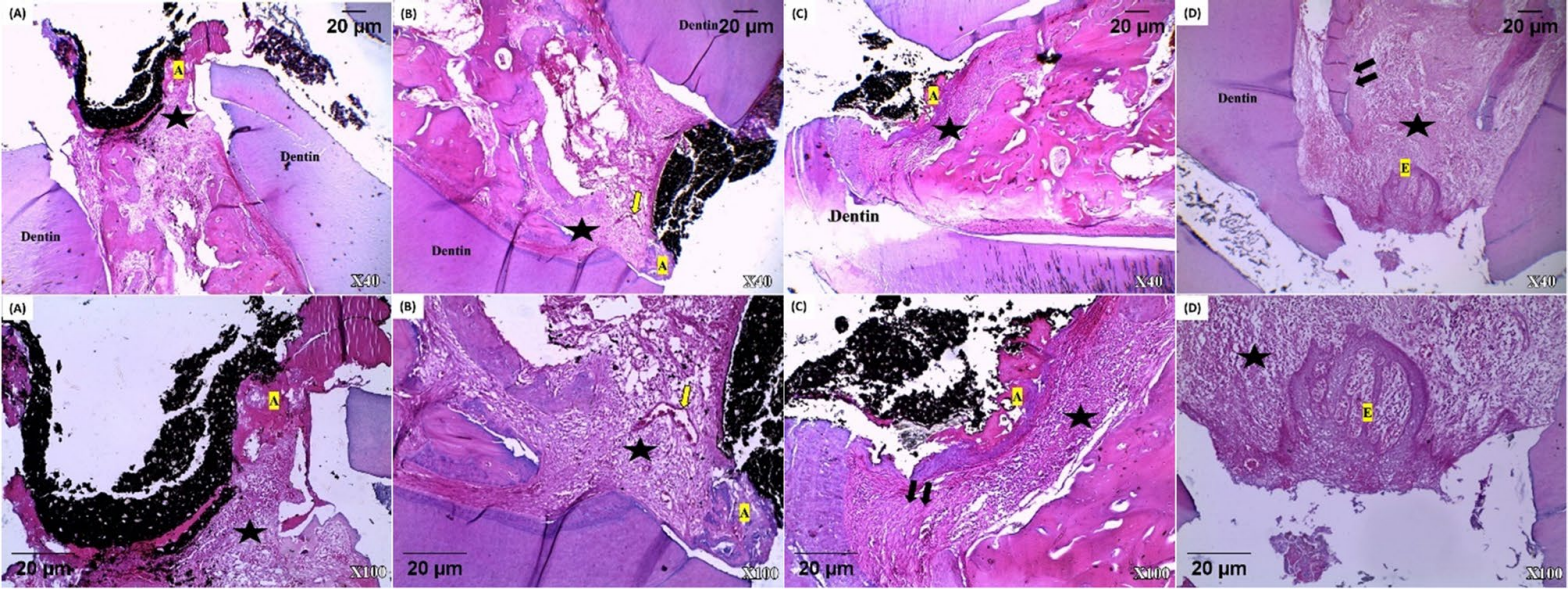
Photomicrographs of all groups after three months. **(A)** PRF group with newly formed hard tissue (A) and surrounding connective tissue showing minimal inflammation (black star) **(B)** Chitosan hydrogel group with newly formed hard tissue (A) with surrounding connective tissue with prominent inflammation (black star) and vasodilation of blood vessels (yellow arrow) **(C)** Bioceramic group with newly formed hard tissue (A) surrounding connective tissue with signs of inflammation (black star) **(D)** Positive control group with epithelial invasion (E) overlying inflamed connective tissue (black star) with bone resorption (black arrows) (H-E; X40-100).

After one month, all samples of the different groups exhibited high percentages of inflammatory cells scores ranged from moderate to severe that decreased in the three months interval as summarized in Table 1. After three months, the control group exhibited the highest percentage scores of inflammation. Moreover, after three months evaluation time, the bioceramic group demonstrated a higher percentage of inflammatory cells scores than PRF group (Fig. 3). In PRF and BC putty groups, there was a decrease in inflammation after three months compared to one month (Fig. 4).

Bone deposition was observed after one month in PRF group in 22.22% of samples with partial hard tissue formation and 44.44% of samples with hard tissue formation sealing the perforation. When chitosan hydrogel was used, 44.44% of samples showed partial hard tissue formation and no samples recorded complete sealing of the perforation. The BC putty group recorded 33.33% of samples with partial hard tissue formation and 22.22% of samples with hard tissue formation sealing the perforation (Figs. 3 and 4).

After three months, the highest percentage of hard tissue formation scores was recorded for PRF group followed by BC putty group. While, in chitosan hydrogel group 44.44% of the samples showed only partial hard tissue formation. In the positive control group, no samples showed newly formed hard tissue as summarized in Table 2.

**Table 1:** Frequencies of inflammatory cells scores and their percentage after one and three months intervals.

Time	Inflammatory cells scores	Groups								Chi-Square	
		G1 (PRF)		G2 (Chitosan Hydrogel)		G3 (Bioceramic)		G4 (Positive Control)			
		N	%	N	%	N	%	N	%	X^2^	***P***-value
1 Month	**Score 0**	0	0.00	0	0.00	0	0.00	0	0.00	5.033	0.540
	**Score 1**	2	22.22	1	11.11	2	22.22	0	0.00		
	**Score 2**	5	55.56	4	44.44	4	44.44	3	33.33		
	**Score 3**	2	22.22	4	44.44	3	33.33	6	66.67		
3 Months	**Score 0**	3	33.33	0	0.00	2	22.22	0	0.00	24.686	0.003* P1 = 0.006* P2 = 0.672 P3 = 0.003* P4 = 0.063 P5 = 0.147 P6 = 0.021*
	**Score 1**	4	44.44	0	0.00	3	33.33	0	0.00		
	**Score 2**	2	22.22	5	55.56	3	33.33	2	22.22		
	**Score 3**	0	0.00	4	44.44	1	11.11	7	77.78		
Chi-Square	**X** ^**2**^	8.939		1.111		3.343		0.277			
	***P*** **-value**	0.030*		0.574		0.342		0.599			

p1 (between G1&G2), p2 (between G1&G3), p3 (between G1&G4), p4 (between G2&G3), p5 (between G2&G4) and p6 (between G3&G4).

Although after one month, there was non significant difference between the different tested groups, the PRF group showed the lowest prevalence of bone resorption followed by BC putty and chitosan hydrogel group respectively with the positive control group showed the highest rate of bone resorption after both time intervals. Bone resorption decreased after three months intervals where no bone resorption scores were recorded for the PRF group, 33.33% of samples showed bone resorption in BC putty group and 55.56% of samples showed bone resorption in chitosan hydrogel group as summarized in Table 3.

**Table 2:** Frequencies of hard tissue formation scores and their percentage after one and three months intervals.

Time	Hard tissue formation	Groups								Chi-Square	
		G1 (PRF)		G2 (Chitosan Hydrogel)		G3 (Bio ceramic)		G4 (Positive Control)			
		N	%	N	%	N	%	N	%	X^2^	***P***-value
1 Month	**Score 0**	3	33.33	5	55.56	4	44.44	9	100.00	15.175	0.019* P1 = 0.076 P2 = 0.604 P3 = 0.011* P4 = 0.324 P5 = 0.023* P6 = 0.031*
	**Score 1**	2	22.22	4	44.44	3	33.33	0	0.00		
	**Score 2**	4	44.44	0	0.00	2	22.22	0	0.00		
3 Months	**Score 0**	0	0.00	5	55.56	3	33.33	9	100.00	22.968	0.001* P1 = 0.007* P2 = 0.162 P3 < 0.001* P4 = 0.162 P5 = 0.023* P6 = 0.011*
	**Score 1**	4	44.44	4	44.44	3	33.33	0	0.00		
	**Score 2**	5	55.56	0	0.00	3	33.33	0	0.00		
Chi-Square	**X** ^**2**^	3.778		0.000		0.343		0.000			
	***P*** **-value**	0.151		1.000		0.842		1.000			

p1 (between G1&G2), p2 (between G1&G3), p3 (between G1&G4), p4 (between G2&G3), p5 (between G2&G4) and p6 (between G3&G4).

In relation to periodontal ligament regeneration, BC putty group with or without PRF matrix at both time intervals exhibited numerous fibroblasts and collagen fiber bundles (Table 4).

In terms of epithelial proliferation, BC putty neither alone nor with PRF matrix exhibited epithelial proliferation. While chitosan hydrogel and positive control groups showed epithelial proliferation at both intervals as shown in Table 5.

**Table 3:** Frequencies of bone resorption scores and their percentage after one and three months intervals.

Time	Bone Resorption	Groups								Chi-Square	
		G1 (PRF)		G2 (Chitosan Hydrogel)		G3 (Bio ceramic)		G4 (Positive Control)			
		N	%	N	%	N	%	N	%	X^2^	*P*-value
1 Month	**Score 0**	7	77.78	3	33.33	5	55.56	2	22.22	6.576	0.087
	**Score 1**	2	22.22	6	66.67	4	44.44	7	77.78		
3 Months	**Score 0**	9	100.00	4	44.44	6	66.67	0	0.00	19.059	< 0.001* P1 = 0.009* P2 = 0.058 P3 < 0.001* P4 = 0.343 P5 = 0.023* P6 = 0.003*
	**Score 1**	0	0.00	5	55.56	3	33.33	9	100.00		
Chi-Square	**X** ^**2**^	2.250		0.234		0.234		2.250			
	***P*** **-value**	0.134		0.629		0.629		0.134			

p1 (between G1&G2), p2 (between G1&G3), p3 (between G1&G4), p4 (between G2&G3), p5 (between G2&G4) and p6 (between G3&G4).

**Table 4:** Frequencies of PDL regeneration scores and their percentage after one and three months intervals.

Time	PDL Regeneration	Groups								Chi-Square	
		G1 (PRF)		G2 (Chitosan Hydrogel)		G3 (Bio ceramic)		G4 (Positive Control)			
		N	%	N	%	N	%	N	%	X^**2**^	*P*-value
1 Month	**Score 0**	6	66.67	9	100.00	6	66.67	9	100.00	9.526	0.023* P1 = 0.058 P2 = 1.000 P3 = 0.058 P4 = 0.058 P5 = 1.000 P6 = 0.058
	**Score 1**	3	33.33	0	0.00	3	33.33	0	0.00		
3 Months	**Score 0**	3	33.33	9	100.00	5	55.56	9	100.00	14.954	0.002* P1 = 0.003* P2 = 0.343 P3 = 0.003* P4 = 0.023* P5 = 1.000 P6 = 0.023*
	**Score 1**	6	66.67	0	0.00	4	44.44	0	0.00		
Chi-Square	**X** ^**2**^	2.000		0.000		0.234		0.000			
	***P*** **-value**	0.157		1.000		0.629		1.000			

p1 (between G1&G2), p2 (between G1&G3), p3 (between G1&G4), p4 (between G2&G3), p5 (between G2&G4) and p6 (between G3&G4).

**Table 5:** Frequencies of epithelial proliferation scores and their percentage after one and three months intervals.

Time	Epithelial Proliferation	Groups								Chi-Square	
		G1 (PRF)		G2 (Chitosan Hydrogel)		G3 (Bio ceramic)		G4 (Positive Control)			
		N	%	N	%	N	%	N	%	X^2^	*P*-value
1 Month	**Score 0**	7	77.78	6	66.67	8	88.89	2	22.22	9.993	0.019* P1 = 0.599 P2 = 0.527 P3 = 0.059 P4 = 0.257 P5 = 0.058 P6 = 0.004*
	**Score 1**	2	22.22	3	33.33	1	11.11	7	77.78		
3 Months	**Score 0**	9	100.00	7	77.78	9	100.00	3	33.33	15.429	0.001* P1 = 0.134 P2 = 1.000 P3 = 0.003* P4 = 0.134 P5 = 0.058 P6 = 0.003*
	**Score 1**	0	0.00	2	22.22	0	0.00	6	66.67		
Chi-Square	**X** ^**2**^	2.250		0.277		1.059		0.277			
	***P*** **-value**	0.134		0.599		0.303		0.599			

p1 (between G1&G2), p2 (between G1&G3), p3 (between G1&G4), p4 (between G2&G3), p5 (between G2&G4) and p6 (between G3&G4).

## Discussion

Biocompatibility and bioactivity of perforation repair materials are important properties that should be considered since these materials come in contact with the vital tissues and can affect the viability of the periradicular cells^1,19^. Therefore, this study was done to evaluate the tissue reaction to different matrix materials used in furcation perforations management.

Dogs are commonly used as experimental animal model in furcation perforation studies. They possess the most suitable model characteristic as they have a comparable apical repair mechanism with humans in shorter duration owing to their high growth rate^20–23^. They also have organic and mineral structure like those of humans and can withstand long periods in the surgical and nonsurgical procedures under general anesthesia^24^.

Trying to adapt the parameters more relevant to the endodontic clinical situation, the teeth involved in the study were isolated using rubber dam sheets and all the operative procedures were done under aseptic conditions to eliminate the effect of contamination in tissue reaction.

The selected size of perforations in the present experiment was 1.4 mm in diameter according to other previous studies^22,25^. All perforations were repaired at the same appointment as the perforations were occured to ensure the most favorable response to perforation repair when they were sealed immediately. This prevented the contamination of the perforation defect from a second intervention and eliminated the need to expose the animal to another general anesthesia.

Although, MTA showed superior properties to other perforation repair materials^17,26,27^, it has some limitations as tooth discoloration, extended setting time and poor manipulation characteristics^28–32^.

Well-Root™ PT is another calcium aluminosilicate material^6^. It has excellent physical and biological properties^33–35^ and proper handlining characteristics therefore, it was used as in perforation repair through this study.

It was suggested that PRF can induce proliferation of fibroblasts and increase collagen formation. It has been used in regeneration of hard and soft tissues during different periodontal surgeries^3,35,36^. For these reasons PRF was used as an internal matrix for repair of furcation perforation in this study.

Chitosan polymers are a natural amino polysaccharides with many characteristics as antimicrobial activity, biocompatibility, and enhancement of cell adhesion, proliferation and cellular differentiation^4,5^. Therefore, in the present study, chitosan hydrogel has been evaluated as a matrix in the repair of the furcation perforation defect.

To establish the normal tissue architecture at the perforation site, keeping in consideration the heterogenous nature of the periodontal and the dentinal wounds dealing with them as separate entities, the use of artificial floor repairing technique was suggested for proper healing after perforation repair^1^. Interference with the periodontal reattachment may occur due to the extruded parts of the repair material into the furcation area. So PRF and chitosan hydrogel were used as a matrix and the premixed Well-Root™ PT was placed on pulpal floor over the internal matrix material^37,38^.

Regarding the inflammatory reaction after one month, the samples of all groups exhibited high percentage inflammatory cell scores that decreased in the three months interval. These findings were explained by the insufficient period for healing to occur^10^ and the inflammatory response of the periodontium to the perforation repair materials^36^. The inflammation to BC putty after one month may be due to its highly alkaline pH, heat produced while setting and the release of cytokines as interleukins-1 and 6^39^. These presenting findings agreed with several other previous studies^1,11,40–42^.

After a three months interval, the positive control group recorded the highest degree of inflammation that may be explained by the absence of repair material in the perforation defect and continued inflammatory reaction. Other groups showed significantly less inflammation due to the sealing ability and the biocompatibility of the materials used to manage the perforation. The present findings were in agreement with several other previous studies^43–48^.

At three months evaluation time showed that BC putty demonstrated higher percentage scores of inflammatory cells than PRF. These findings may be explained by the autogenous origin and anti-inflammatory properties of PRF^49^. In addition to that, the role of PRF in antimicrobial host defense^50^.

In PRF and BC putty groups, inflammation was reduced after three months compared to one month with similar reasons reported before. The reduction in percentage scores of inflammatory cells after three months compared to one month was statistically significant for the PRF group. These finding was in agreement with Tawfik et al.^36^. who reported that when PRF was used as a matrix and MTA as a base on pulpal floor, there was a significant decrease in inflammation. Similar findings were found with Al-Adimi et al.^10^ who used MTA with calcium sulphate as artificial floor and recorded initial high inflammatory response at one-month interval that reduced gradually in the subsequent three and six months intervals.

In relation to chitosan hydrogel group at three months interval, significantly higher inflammatory reaction than PRF group was recorded. This may be attributed to its low mechanical strength as supported by Liu et al.^51^. Another alternative explanation for the increased percentages of inflammatory cell scores may be due to its cationic nature of amino groups that interact with the anionic extracellular matrix molecules such as glycosaminoglycans electrostatically. Furthermore, the interactions with cytokines and growth factors can affect cellular process^52,53^.

Additionally, chitosan hydrogel revealed lower percentages of inflammatory cell scores than the positive control group highlighting its high biocompatibility and proper biodegradability. The findings were in agreement with Alsawah et al.^38^.

Considering hard tissue formation, when comparing all groups at one and three months intervals all tested repair materials (PRF, chitosan hydrogel and BC putty) showed significantly better hard tissue formation compared to the positive control group. However, PRF group showed higher scores of new hard tissue formation than that of BC putty and chitosan hydrogel group. This could be due to the healing nature of PRF^54^ that was supported by Tawfik et al.^36^.

On the other hand, it was found that injectable chitosan hydrogel increased the release of BMP-2 and osteoblasts differentiation in another research^5^ which was supported by the current results where chitosan hydrogel showed significantly hard tissue formation compared to the control group. Additionally, this may be attributed to that chitosan hydrogel may accelerate the rate of new bone apposition due to its biocompatibility and enhancement of cell adhesion, proliferation and cellular differentiation^4,5^. Furthermore, the presence of β-GP in chitosan hydrogel has been shown to be an osteogenic action^12^. These findings were in agreement with Alsawah et al.^38^. when they used chitosan scaffold for perforation repair.

Furthermore, the efficacy of PRF and chitosan hydrogel in hard tissue formation may be due to their ability to resist the extrusion of the repairing material into the inter-radicular space efficiently that subsequently inhibited the further irritation of the extruded material and played an important role in deposition of hard tissue. Moreover, several authors revealed that their rate of resorption coincided with new hard tissue formation, which in turn aided in tissue regeneration and excluded epithelium from the area of bone formation^55,56^. These findings were supported by previous studies^1,57–60^.

The present study showed that the premixed bioceramic repairing material used alone without an internal matrix was more effective than chitosan hydrogel group in hard tissue formation. These findings may be explained by BC putty repairing material is characterized by its alkaline pH mediated by calcium hydroxide release during setting promoting hard tissue^6,61^. This was in agreement with the studies conducted by several authors^22,41,62–65^. This was supported by Al-Daafas et al.^11^ who proved that MTA has much greater activity in formation of new hard tissue over MTA used with calcium sulfate matrix. In positive control group, no new hard tissue was formed due to the presence of persistent inflammation.

After three months, the new hard tissue formation increased, and bone loss decreased for all repair materials. This agreed with the findings of other studies^1,11,38,41,42^.

Regarding the parameter of bone resorption, higher frequencies were shown in positive control groups compared to the other experimental groups. This possibly is due to the great inflammation that occurred from the untreated defect. Moreover, the present findings revealed a decrease in bone loss by time in experimental groups where BC putty used with or without PRF and chitosan hydrogel internal matrices. These findings agreed with Alhadainy et al.^1^.

The initial bone resorption that was recorded at one month interval may be related to the heat occurred during creating the perforation. This may cause resorption of bone and cementum^10^.

Moreover, PRF group resulted in a statistically significant lower bone resorption than chitosan hydrogel group after three months evaluation period as PRF can reduce the inflammation and increase release of growth factors^35^. Furthermore, the BC putty group recorded higher bone resorption than PRF group which can be explained by material extrusion as matrix was used. These results agreed with Tawfik et al.^36^.

These findings were supported by Al-Daafas et al.^11^ who sated that using of a matrix below repair material in management of furcation perforation resulted in reduction of tissue inflammation^22,57^.

Also, an increase in bone resorption in chitosan hydrogel group compared to both PRF and BC putty groups after one and three months intervals was demonstrated, and it was significant when compared to PRF after three months intervals. This finding may be explained by the cationic nature of chitosan making it highly reactive and could interact physically to other charged particles compromising its efficacy^52^. Moreover, chitosan has the ability to chelate metals that may result in reducing enzyme activity and this was associated with bone resorption^66^.

These results agreed with Alsawah et al.^38^. who also detected osteoclastic activity in 25% of samples at one and three months intervals in the chitosan scaffold group. These findings also agreed with previous studies^67,68^.

At the present study, periodontal ligament regeneration was observed in BC putty group with or without PRF matrix at both time intervals owing to the low inflammation encountered^69^. These results were in agreement with Al-Adimi et al.^10^ and supported by the finding of Silva et al.^70^.

The potential of PRF to induce PDL regeneration at one and three-months intervals may be due to the less inflammation elicited with it as supported by Tawfik et al.^36^. The placement of a matrix in the perforation defect maintained the repair material in situ permitting more time for healing to occur^1,11,55^. Regarding BC putty efficiency in PDL regeneration, it may be related to that it allowed the cementum formation and allowed the regeneration of the PDL. The current results were supported with other findings of several authors^1,11,47,55^.

In contrast, the current histological evaluation revealed that neither fibroblasts nor collagen fiber bundles were seen with chitosan hydrogel and positive control groups owing to the higher levels of inflammatory reaction and resorption occurred with these groups. Supporting the results of the present study, similar findings were obtained by Abboud et al.^47^.

In terms of epithelial proliferation, BC putty with or without PRF showed no epithelial invasion at 3 months interval. This is attributed to its high biocompatibility and sealing ability^10,41^. While chitosan hydrogel and positive control groups showed epithelial proliferation at both intervals. The invasion of epithelium to the perforation areas was reported also in previous studies^1,12^. The epithelial invasion occurred may be explained by the closeness of the dog’s teeth cementoenamel junction to the furcation area^11^.

Several limitations of the present study should be acknowledged. One important limitation is that although a post-hoc power analysis was conducted, the relatively small sample size means that the study was not adequately powered to detect small-to-moderate effect sizes. While the results were consistent across the tested groups, subtle differences may not have reached statistical significance. Future studies with larger sample sizes are therefore needed to confirm and expand upon the current findings.

Another limitation is that this study did not include PRF-only or chitosan-only control groups. The rationale was that both PRF and chitosan, when used alone without supporting base material, exhibit a wet nature that reduces their mechanical stability and negatively affects the final restorative material. For this reason, their isolated application was not feasible within the current experimental design. However, the absence of these groups remains an important limitation, as it restricts the ability to clearly distinguish matrix-specific effects from those combined effects of the matrix and BC putty as a base. Future studies incorporating such control groups are warranted to better elucidate their distinct and synergistic roles.

A further limitation relates to the use of an acute perforation model, which offers a standardized and reproducible approach for experimental assessment. However, it does not fully replicate the clinical situation of chronic furcation perforations, which are often associated with persistent inflammation, bacterial contamination, and granulation tissue formation. These chronic features may significantly influence the healing response and the performance of the tested materials. Therefore, extrapolation of the present findings to long-standing clinical cases should be made with caution. Future studies incorporating chronic perforation models will be essential to provide a more clinically relevant understanding of treatment outcomes.

In addition, while the subjective histologic scoring (though blinded) offers valuable insights into the nature and quality of tissue response, it represents only a snapshot at specific time intervals. Complementary methods such as micro-CT, molecular analysis of inflammatory and regenerative markers, and long-term functional assessments could provide a more comprehensive understanding of the biological events underlying the observed healing patterns.

Lastly, the variability in PRF preparation is also considered a limitation in this study. Despite these limitations, the present work provides important preliminary data that may serve as a foundation for further investigations.

## Conclusions

Considering the limitations of this study, BC putty exhibited favorable outcomes when applied without chitosan hydrogel as an internal matrix for the repair of furcal perforations. In addition, the combination of PRF and BC putty appears to be a reliable option and PRF may be considered a material of choice for internal matrix application in the management of furcation perforations.

## Data Availability

All data used is readily available from the corresponding author when requested.

## Abbreviations

ARRIVE: The animal research: Reporting in vivo experiments
BC: Premixed bioceramic
CH: Chitosan
GP: Glycerophosphate
MTA: Mineral trioxide aggregate
REC: Research Ethics Committee
PRF: Platelet rich fibrin
PDL: Periodontal ligament
